# 2364. Predictive Ability of mRNA COVID-19 Vaccines Against COVID-19 Disease Severity

**DOI:** 10.1093/ofid/ofad500.1985

**Published:** 2023-11-27

**Authors:** Ashley Lew, Ashley Tippett, Luis W Salazar, Laila Hussaini, Chris Choi, Khalel De Castro, Elizabeth G Taylor, Olivia Reese, Humerazehra Momin, Caroline R Ciric, Amrita Banerjee, Amy E Keane, Laura A Puzniak, Robin Hubler, Srinivas Valluri, Timothy L Wiemken, Benjamin Lopman, Nadine Rouphael, Satoshi Kamidani, Evan J Anderson, Christina A Rostad

**Affiliations:** Emory University School of Medicine, Atlanta, Georgia; Emory University, Atlanta, Georgia; Emory University, Atlanta, Georgia; Emory Univeristy, Atlanta, Georgia; Emory University, Atlanta, Georgia; Emory University School of Medicine, Atlanta, Georgia; Emory University School of Medicine, Atlanta, Georgia; Emory University, Atlanta, Georgia; Emory University School of Medicine, Atlanta, Georgia; Emory University, Atlanta, Georgia; Emory University, Atlanta, Georgia; Emory University, Atlanta, Georgia; Pfizer Inc., Collegeville, Pennsylvania; Pfizer Inc., Collegeville, Pennsylvania; Pfizer Inc, New York, New York; Pfizer Inc, New York, New York; Rollins School of Public Health | Emory University, Atlanta, Georgia; Emory University School of Medicine, Atlanta, Georgia; Emory University School of Medicine and Children's Healthcare of Atlanta, Atlanta, Georgia; Moderna, Inc., Atlanta, Georgia; Emory University School of Medicine and Children's Healthcare of Atlanta, Atlanta, Georgia

## Abstract

**Background:**

Prior studies demonstrated the vaccine effectiveness and safety of mRNA COVID-19 vaccines, but additional data is needed regarding the effects of timing and number of doses on disease severity. This study determined predicted protection against severe COVID-19 by number of vaccine doses.

**Methods:**

We enrolled adults hospitalized with acute respiratory infection (ARI) and/or related diagnoses at two Emory University hospitals from May 2021 – Aug 2022. This analysis included COVID-19 positive patients among unvaccinated and 2 or 3 doses of an mRNA vaccine. Vaccinations ≤ 14 days prior to admission were excluded. Medical and social histories were obtained from interviews, medical records, and the state vaccine registry. We used stepwise logistic regression to determine dose-specific odds ratios (OR) against severe outcomes (pneumonia (PNA), length of hospital stay (LOS) ≥ 4 days, ICU admission, mechanical ventilation, and death). Analysis was performed using SAS v9.4 software.

**Results:**

Of the 1,677 total enrollments, 850 were positive for COVID-19. Another 168 were excluded due to lack of vaccine records or other vaccine dosages, 682 were eligible for analysis. Compared to those unvaccinated, vaccinated participants were older, male, and unemployed or on disability. Those with three doses obtained a higher education level than unvaccinated participants. Individuals with comorbidities – specifically blood disorders, chronic kidney disease, and immunocompromised – were more often vaccinated. When controlling for race, age, and employment, the odds of PNA (OR 0.5 (0.34, 0.74)) and ICU admission (0.61 (0.39, 0.97)) were less for those with two doses than those with none; whereas those with three mRNA doses had less odds of having PNA (OR 0.26 (0.15, 0.46) and LOS ≥ 4 days (OR 0.44 (0.25, 0.76)). Predicted protection against severe outcomes persisted 6 months from last dose for PNA, LOS ≥ 4 days, and ICU admission (Table 1).

**Table 1.**
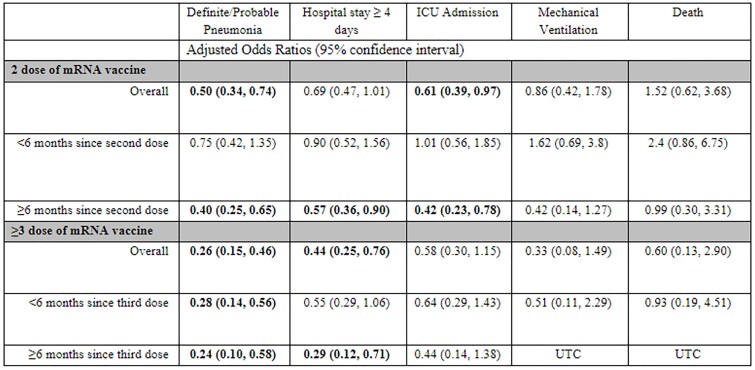
Adjusted odds ratios of mRNA SARS-CoV-2 vaccination by dose number and time since administration against severe disease outcomes in those SARS-CoV-2 positive. OR adjusted for race, age and employment. Unable to calculate (UTC) due to having no patients with the outcome during specified vaccination timeframe and dose count.

**Conclusion:**

Among COVID-19 positive adults hospitalized with ARIs, both two and three doses of a COVID-19 mRNA vaccine predicted protection against severe COVID-19 outcomes, with durability lasting more than 6 months. Future studies should explore the role of additional boosters and vaccine regimens in the prevention of severe COVID-19.

**Disclosures:**

**Laura A. Puzniak, PhD. MPH**, Pfizer, Inc.: Employee|Pfizer, Inc.: Stocks/Bonds **Robin Hubler, MS**, Pfizer, Inc.: Employee|Pfizer, Inc.: Stocks/Bonds **Srinivas Valluri, PhD**, Pfizer Inc: Pfizer Employee and hold Pfizer stocks/options|Pfizer Inc: Ownership Interest|Pfizer Inc: Stocks/Bonds **Timothy L. Wiemken, PhD**, Pfizer Inc: Employee|Pfizer Inc: Stocks/Bonds **Benjamin Lopman, PhD**, Epidemiological Research and Methods, LLC: Advisor/Consultant|Hillevax, Inc: Advisor/Consultant **Nadine Rouphael, MD**, Icon, EMMES, Sanofi, Seqirus, Moderna: Advisor/Consultant **Satoshi Kamidani, MD**, CDC: Grant/Research Support|Emergent BioSolutions: Grant/Research Support|NIH: Grant/Research Support|Pfizer Inc: Grant/Research Support **Evan J. Anderson, MD**, GSK: Advisor/Consultant|GSK: Grant/Research Support|Janssen: Advisor/Consultant|Janssen: Grant/Research Support|Kentucky Bioprocessing, Inc.: Safety Monitoring Board|Moderna: Advisor/Consultant|Moderna: Grant/Research Support|Moderna: Currently an employee|Moderna: Stocks/Bonds|Pfizer: Advisor/Consultant|Pfizer: Grant/Research Support|Sanofi Pasteur: Advisor/Consultant|Sanofi Pasteur: Grant/Research Support|Sanofi Pasteur: Safety Monitoring Board|WCG/ACI Clinical: Data Adjudication Board **Christina A. Rostad, MD**, BioFire Inc.: Grant/Research Support|GlaxoSmithKline Biologicals: Grant/Research Support|Janssen: Grant/Research Support|MedImmune LLC: Grant/Research Support|Meissa Vaccines, Inc.: RSV vaccine technology|Merck & Co., Inc.: Grant/Research Support|Micron Technology, Inc.: Grant/Research Support|Moderna, Inc.: Grant/Research Support|Novavax: Grant/Research Support|PaxVax: Grant/Research Support|Pfizer, Inc.: Grant/Research Support|Regeneron: Grant/Research Support|Sanofi Pasteur: Grant/Research Support

